# Magnetic resonance imaging of inflammatory pseudotumor of the liver: a 2021 systematic literature update and series presentation

**DOI:** 10.1007/s00261-022-03555-9

**Published:** 2022-06-01

**Authors:** Linda Calistri, Davide Maraghelli, Cosimo Nardi, Sofia Vidali, Vieri Rastrelli, Laura Crocetti, Luigi Grazioli, Stefano Colagrande

**Affiliations:** 1 Department of Experimental and Clinical Biomedical Sciences, Radiodiagnostic Unit n. 2, University of Florence - Azienda Ospedaliero-Universitaria Careggi, Largo Brambilla 3, 50134 Florence, Italy; 2 Clinical and Translational Science Research Department - Division of Interventional Radiology, Cisanello University Hospital, Bldg 30, Via Paradisa 2, 56124 Pisa, Italy; 3 Department of Radiology, University of Brescia “Spedali Civili”, P. le Spedali Civili 1, Brescia, Italy

**Keywords:** Magnetic resonance imaging, Inflammatory pseudotumor of the liver, IgG4-related disease, Focal liver lesion, Targetoid aspect

## Abstract

**Purpose:**

Inflammatory pseudotumors of the liver (IPTL) are not exceptional benign lesions with various etiologies, histology, and imaging appearances. The incomplete knowledge of this pathology and the wide polymorphism sometimes resembling malignancy often induce long and expensive diagnostic flow, biopsy and occasionally unnecessary surgery. We propose a systematic revision of MRI literature data (2000–2021) with some narrative inserts and 10 new complete MRI cases, with the aim of organizing the data about IPTL and identifying some typical features able to improve its diagnosis from imaging.

**Methods:**

We performed a systematic revision of literature from 2000 to 2021 to obtain MRI features, epidemiological, and clinical data of IPTL. The basic online search algorithm on the PubMed database was “(pseudotumor) AND (liver) AND (imaging).” Quality assessment was performed using both scales by Moola for case report studies and by Munn for cross-sectional studies reporting prevalence data. A case-based retrospective study by collecting patients diagnosed with IPTL from three different university hospitals from 2015 to 2021 was done as well. Only cases with MR examinations complete with T1/T2/contrast-enhanced T1/Diffusion-Weighted (W) images and pathology-proven IPTL were selected.

**Results:**

After screening/selection 38 articles were included for a total of 114 patients. In our experience we selected 10 cases for a total of 16 IPTLs; 8 out of 10 patients underwent at least 1 MRI follow-up. Some reproducible and rather typical imaging findings for IPTL were found. The targetoid aspect of IPTL is very frequent in our experience (75% on T1W, 44% on T2W, 81% on contrast-enhanced T1W (at least one phase), 100% on Diffusion-W images) but is also recurrent in the literature (6% on T1W, 31% on T2W, 51% on CE-T1W (at least one phase), 18% on Diffusion-W images, and 67% on hepatobiliary phase). In our experience, Apparent Diffusion Coefficient map values were always equal to or higher than those of the surrounding parenchyma, and at MRI follow-up, nodule/s disappeared at first/second control, in six patients, while in the remaining 2, lesions persisted with tendency to dehydration.

**Conclusion:**

A targetoid-like aspect of a focal liver lesion must raise diagnostic suspicion, especially if IgG4-positive plasma is detected. MRI follow-up mainly shows the disappearance of the lesion or its reduction with dehydration.

**Graphical abstract:**

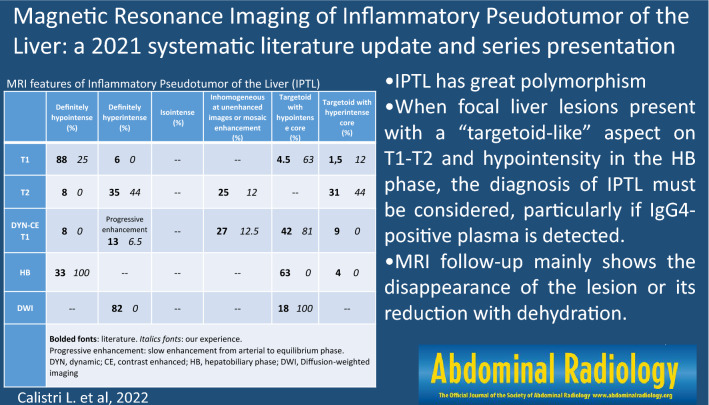

**Supplementary Information:**

The online version contains supplementary material available at 10.1007/s00261-022-03555-9.

## Introduction

Inflammatory pseudotumor of the liver (IPTL), first described in 1953 by Pack and Baker, is more commonly found in young Asian adults with a male-to-female ratio between 3:1 and 8:1 [1]. Four groups of etiologies have been associated with its development: infectious (mainly bacterial), immunologic, allergic, or neoplastic causes [1]. In some patients, IPTL arises after trauma or surgery, and it can be associated with other inflammatory lesions, such as sclerosing cholangitis, retroperitoneal fibrosis, and autoimmune diseases [2, 3]. Histologically, IPTL presents a fibrous-inflammatory infiltrate of variable composition. In 1978, Someren divided IPTL into 3 subtypes, characterized by (1) prominent histiocytic component, (2) prevalent plasma cell component, and (3) markedly sclerotic features [4]. More recently, a close relationship between IgG4-related immune reactions and inflammatory pseudotumors was suggested, and Zen et al. classified IPTLs into the fibro-histiocytic type (xanthogranulomatous inflammation) and lympho-plasmacytic type with numerous IgG4-positive plasma cells in the biopsy sample and high-serum IgG4 levels [5]. The US, CT, and MR appearance of IPTL seem extremely variable [1]. It has a good prognosis; regression can occur spontaneously or could be achieved with anti-inflammatory/steroid therapy [6]. This lesion is rare but not exceptional and with increasing incidence, perhaps for the greater accuracy of the imaging technologies. Since 1990, the number of cases reported in PubMed has doubled every 10 years. Moreover, it is poorly understood, and the vast majority of articles dealing with IPTL are isolated case reports; very few manuscripts accurately describe the MR features of IPTL with a comprehensive analysis of signal intensity (SI) at unenhanced and enhanced imaging [7–9], and only a few evaluate the evolution of IPTL over time through MR follow-up [10–16]. This fragmented knowledge and the wide polymorphism that can resemble malignancy often induce long and expensive diagnostic flow and sometimes unnecessary surgery.

Our work includes a systematic revision of MR Imaging (MRI) literature data (2000–2021) with some narrative inserts, and in addition describes 10 new complete MRI cases, with the aim of organizing the data about IPTL and, if possible, identifying some typical/indicative features able to improve its diagnosis from imaging.

## Materials and methods

### Study design

We performed a systematic review of the literature from January 2000 to May 2021 to obtain a large consensus of MRI appearances and epidemiological and clinical data of IPTL. We also performed a case-based retrospective study by collecting MRI appearances and epidemiological and clinical data of patients diagnosed with IPTL between 2015 and 2021 from three different institutions.

### Literature data

#### Search strategy

The basic online search algorithm on the PubMed database was “(pseudotumor) AND (liver) AND (imaging)” (time filter from 2000 to 2020—in addition, five manuscripts from the first 5 months of 2021). The search was conducted in 2021 May by DM. Concerning to the time filter, we decided to start in 2000, because Diffusion-Weighted Imaging (DWI) has been used for the evaluation of liver parenchyma since that year [17].

#### Study selection

**Fig. 1:**
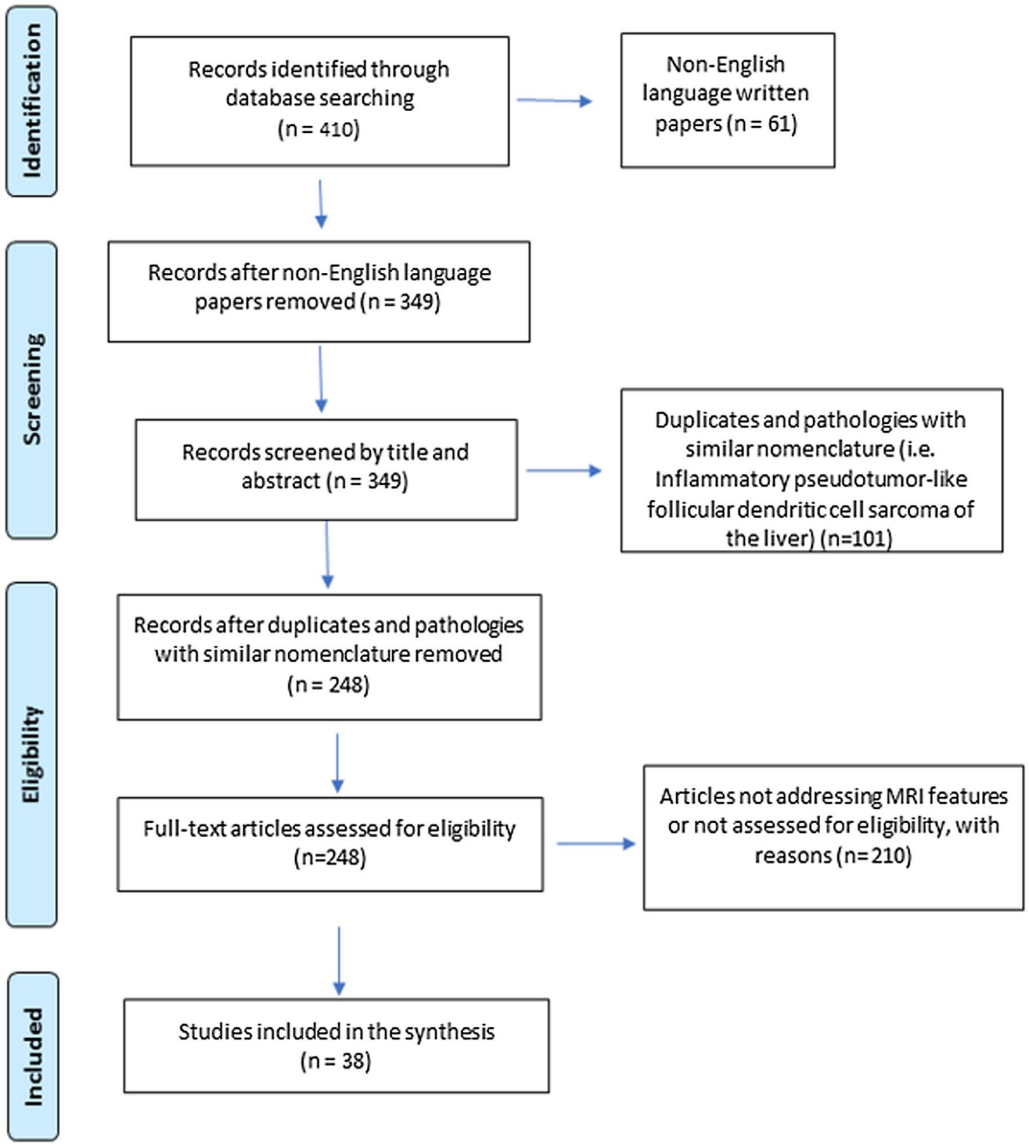
Flow diagram illustrating the selection of papers extracted from literature

The results from the searches were evaluated for selection based on their titles and abstracts. The abstracts/full texts of all the studies were compared to the inclusion criteria. The selection of documents that met the inclusion criteria was independently conducted by two reviewers (DM, SV) and supervised by SC. The study search and selection process are schematized in the flow diagram shown (Fig. 1). Inclusion criteria included written English language papers, the presence of imaging information (MRI including T1/T2-Weighted (T1/T2W) sequences and/or dynamic contrast-enhanced T1 Weighted (CE-T1W) imaging and/or DWI), imaging figures, and epidemiological and clinical data of patients with pathology-proven IPTL. Pre-published previews of accepted articles were not considered for inclusion. Non-English language written works, papers with no clinical case presentation (i.e., meta-analyses, reviews, comments), and those with no MRI imaging data and no pathology information were excluded. Peribiliary forms were not considered in our study. References of included works were also evaluated. The data that support the findings of this study are available from the corresponding author upon reasonable request.

#### Quality assessment

Quality assessment was performed using two different evaluation scales: the quality assessment scale by Moola et al. for case report studies and the quality assessment scale by Munn et al. for cross-sectional studies reporting prevalence data [18, 19].

#### Data extraction

Full texts from included manuscripts were analyzed. The extraction of data was performed using a scheme designed to address the search questions consisting of the article title, patients’ demographic, and clinical characteristics; SI on T1/T2/CE-T1W images, DWI and apparent diffusion coefficient (ADC) maps; eventual pathology or clinical proof of IPTL; serum IgG4 levels and/or the presence of IgG4-positive plasma cells in the inflammatory infiltrate of IPTL; and lesions’ location and their relation with bile and blood vessels. It should be specified that if the localization of IPTL extends to more than one segment, all the segments mentioned in the text were considered. Therefore, a lesion occupying 3 segments is counted 3 times, once for each segment. To calculate the percentage of the lobe localization, we have not only considered the IPTLs of which we are aware only of the lobe, but also the IPTLs with known segment localization. These latter are grouped in the lobes to which they belong. We have considered segments 1–4 as left lobe, while segments 5–8, as right lobe. The extracted data were collected in a Microsoft Excel spreadsheet. Inaccuracy in document inclusion and data extraction was limited by the work of three reviewers (VR, LCa, CN). Disagreements between reviewers were resolved with collegial discussion.

#### Data synthesis

Data extracted from included studies were then pooled together in order to provide a descriptive synthesis of demographic and clinical-radiological features of interest. Continuous variables were summarized with means and ranges, while dichotomous and categorical variables with frequencies and relative percentages.

### Our experience

#### Patients and methods

**Fig. 2:**
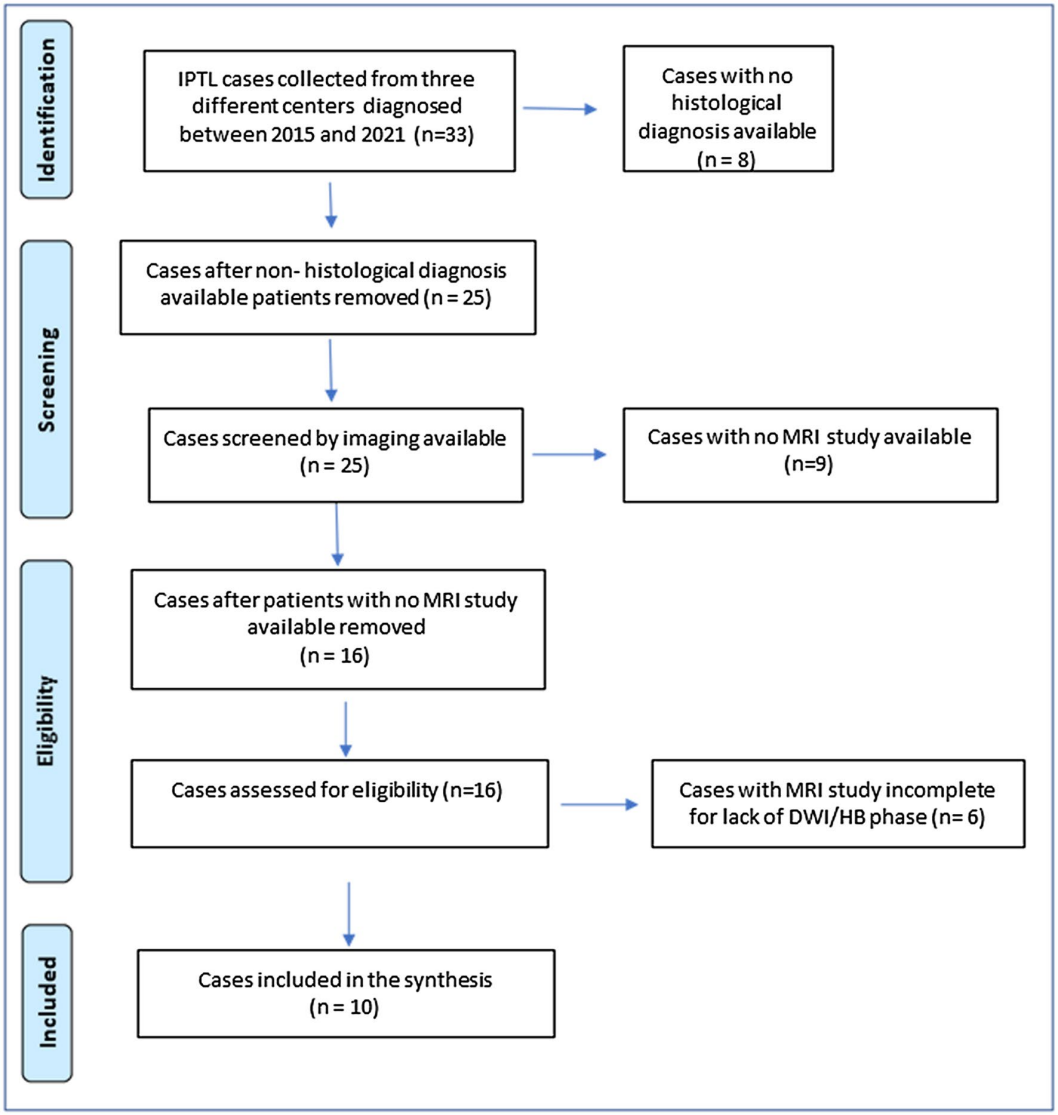
Flow diagram illustrating the selection of cases from our databases

This was a cross-sectional, retrospective study on MRI features of IPTL. Cases were collected from three university hospitals imaging departments (“Careggi University Hospital”, Florence—“Cisanello University Hospital”, Pisa—“Spedali Civili University Hospital”, Brescia) from 2015 to 2021. The patient search and selection process are schematized in the flow diagram shown in Fig. 2. Inclusion criteria included MR examinations complete with T1/T2/CE-T1/DW images and pathology-proven IPTL. Incomplete MR examinations and uncertain diagnosis of IPTL not confirmed with histopathology were excluded. Peribiliary forms were not considered in our study. Both MR examinations at baseline and follow-ups were evaluated, if available. Patient demographic and clinical characteristics and data regarding positivity for IgG4 were collected. As in literature data extraction (see above), lesions’ location and their relationship with bile and blood vessels were also taken in account. All patient imaging data were analyzed by a consensus of two radiologists experienced in abdominal imaging (SC, LG). Institutional review board approval and patient consent were not required for this retrospective study because patient privacy was maintained (DICOM files anonymized before their extraction) and patient care was not impacted.

#### Imaging protocols

The MR examinations were performed with three different tomographs: a 1.5-T MR body scanner (Aera and Avanto at Careggi University Hospital and Spedali Civili University Hospital, respectively; Siemens Medical Systems, Erlangen, Germany) with a maximum gradient strength of 45 mT/m, the peak slew rate of 200 mT/m/ms, and a 4-channel phased array body coil and a 1.5-T MR body scanner (GE; Signa Excite HDTX Twin Speed at Cisanello University Hospital; USA, Wisconsin, Milwaukee) with a maximum gradient strength of 30 mT/m, the peak slew rate of 120 mT/m/ms, and a 4-channel phased array body coil. The acquisition protocol and parameters applied in every institution are schematized in Table 1. Fat suppression was obtained by spectral presaturation inversion recovery (SPAIR). Isotropic motion-probing gradients were applied. The ADC map was automatically generated from all the diffusion weightings and calculated on a picture archiving and communication system workstation. Enhanced MRI was obtained by administration of a dose of 0.025 mmol/kg of body weight gadoxetic acid disodium (EOB-Gd, Primovist; Bayer Schering, Berlin, Germany) followed by 20 mL of sterile saline solution injected via an antecubital vein with a flow rate of 1 mL/s using a power injector (Veris, Medrad, Pittsburgh, PA). A 3D gradient-recalled echo T1W breath-hold sequence with fat suppression was acquired before and after the intravenous administration of the liver-specific contrast agent (CA) with a standard delay technique during the arterial (25–35 s), portal (70 s), and equilibrium (180 s) phases of the dynamic study and 20 min after the bolus injection in the hepatobiliary phase (HBP).

**Table 1:** Unenhanced MRI protocol with acquisition parameters

		Axial Gradient echo T1W in/out of phase	Axial FS TSE T2W	Axial HASTE T2W	Coronal HASTE T2W	3D Axial FS Gradient-recalled echo T1W	Axial EPI SE DW 0–750 mm^2^/s
TR, ms	AERA-AVANTO (Siemens) SIGNA (GE)	170/490	1000/5254	327	1000/5294	3.26/4.4	6000
TE, ms	AERA-AVANTO (Siemens) SIGNA (GE)	1.59/3.26	85/106	96	92/97.50	1.5/2	63
FOV, mm, AP-RL	AERA-AVANTO (Siemens) SIGNA (GE)	300–420	300–420	390	300–420	300–420	420
Matrix	AERA-AVANTO (Siemens) SIGNA (GE)	104 × 256 320 × 320	104 × 256 384 × 384	104 × 256 384 × 384	104 × 256 320 × 320	104 × 256 320 × 320	104 × 256 320 × 320
Thickness, mm	AERA-AVANTO (Siemens) SIGNA (GE)	4–6	4–6	4–6	4–6	2.3–3	4–6
Fat sat method	AERA-AVANTO (Siemens) SIGNA (GE)	Dixon	SPAIR ASPIR	N/A	N/A	SPAIR ASPIR	SPAIR ASPIR

*T1W* T1 weighted, *FS* fat saturation, *TSE* turbo spin echo, *T2W* T2 weighted, *HASTE* half-Fourier acquisition single-shot turbo spin echo, *3D* three-dimensional, *EPI* echo planar imaging, *SE* spin echo, *DW* diffusion weighted, *TR* repetition time, *TE* echo time, *FOV* field of view, *AP* anterior–posterior, *RL* right–left, *SPAIR* spectral attenuated inversion recovery, *ASPIR* adiabatic spectral inversion recovery, *N/A* not applicable

## Results

### Literature data analysis

From the initial search, 420 studies were collected in the period 2000–2020. In addition, five manuscripts were added from January to May 2021. After excluding non-English language written papers, screening, and eligibility, 38 articles were included (Fig. 1): 4 cross-sectional studies reporting prevalence data and 34 case reports, for a total of 114 patients [1, 2, 7–16, 20–45]. The number of patients in the 4 cross-sectional studies was 13, 19, 23, and 25 [7, 8, 31, 45]. The study by Park [31] reported 45 IPTL patients; however, only 23 of them had been studied with MRI and therefore, only these were considered in our paper. According to the evaluation scale by Moola [18], of the 34 case reports, 10 met all the criteria, considering that the parameter on adverse events/unanticipated events was not deemed applicable for any article (no adverse event or unanticipated event occurred). Almost all the case reports presented a good description of the current clinical condition, diagnostic tests, and treatments performed but did not report the complete characteristics of the lesions: some studies reported only the features of the unenhanced examination or only those of the CE sequences, with or without HBP and DWI. The most missing data are concentrated on demographic characteristics and patient history. The insufficient data mainly concern race, medical history, previous treatment, medications routinely used by the patient, and family history. According to the evaluation scale by Munn [19], of the 4 manuscripts that fall into the cross-sectional study category, one fully meets all the criteria. The other 3 partially lack some data, such as morbidities, medications, and other potentially influential factors. Quality assessment tables are available in Supplemental 1.

### Patients’ selection in our experience

Initially, 33 cases of IPTL were retrospectively collected from the three aforementioned centers. Of these, 8 were excluded because a histopathological diagnosis was not available, 9 were further ruled out as not even an MR examination had been performed, but only US/CT evaluations, and 6 were excluded because the MR assessment was incomplete (lack of DWI and/or HBP), so 10 patients met the inclusion criteria (Fig. 2).

### Epidemiological and clinical features

In the literature, epidemiological and clinical data were not always available (Table 2). Regarding mean age and sex, out of 91 patients with available data, 62 were male (68%) and 29 females (32%) with a mean age of 54 years (age range 2–82 years), prevalently Asians (91%). A total of 140 pathologically proven IPTLs were detected: single in 97 cases and multiple in 17 cases. The mean maximum diameter of the IPTLs was 33.8 mm (size range 8–200 mm). Symptoms were present in 54% of cases; 46% of the patients were completely asymptomatic with accidental detection. The most frequent symptom was upper abdominal quadrant pain (64%), followed by fever (51%), and the most frequent associated condition was chronic liver disease of various origins, found in 11 patients. IgG4-positive plasma cell infiltrate was present in 46% of patients in whom this evaluation was performed (41 patients, predominantly males with a solitary nodule; IgG4 serum levels not available).

**Table 2:** Epidemiological and clinical features of both literature and our cases

Patients	Literature (*n* = 114)	Our cases (*n* = 10)
Sex	*N* = 91; 62 m (68%) 29 f (32%)	*N* = 10; 6f (60%) 4 m (40%)
Mean age	*N* = 91; 54 yo (S.D. 14.9)	*N* = 10; 56 yo (S.D. 12.0)
Race	*N* = 114; 104 Asians (91%) 10 Caucasians (9%)	*N* = 10; Caucasians (100%)
Present/absent symptoms	*N* = 72; 39 present (54%) 33 absent (46%)	*N* = 10; 2 present (20%) 8 absent (80%)
More frequent symptoms (more symptoms are often present in the same patient, e.g., fever + pain)	*N* = 39; 25 upper abdominal pain (64%), 20 fever (51%), 2 jaundice (5%), 2 asthenia (5%)	*N* = 2; 1 fever (50%), 1 upper abdominal pain (50%), 1 palpable abdominal mass (50%)
Presence of associated conditions	*N* = 51; 22 yes (43%); 29 no (57%)	*N* = 10; 5 yes (50%); 5 no (50%)
More frequent associated conditions	*N* = 22; 4 alcoholism / alcoholic cirrhosis (18%), 4 unspecified chronic liver disease (18%), 3 HBV + (13%), 3 history of rectum neoplasia (13%)	*N* = 5; 3 fatty liver disease (60%), 2 history of lymphoma (40%)
Mean maximum diameter of IPTL	*N* = 131; 33.8 mm (S.D. 21.7)	*N* = 10; 40.0 mm (S.D. 47.1)
IGg4-positive plasma cell infiltrate	*N* = 41; 19 positive patients (46%), 22 negative patients (54%)	*N* = 10; 2 positive patients positive (20%), 8 negative patients (80%)
IGg4-serum levels (> 135 mg/dL)	IgG4 serum levels not available^a^	*N* = 2

*n* total number of patients, *N* number of patients for whom the data are available, *HBV* hepatitis B virus, *m* male, *f* female, *yo* year old, *IPTL* inflammatory pseudotumor of the liver, *S.D.* standard deviation

^a^85% according to [51], not included in the systematic review

In our experience, we collected records from 10 patients with IPTL (4 males, 6 females; mean age 56 years, age range 38–77 years), all Caucasians. The diagnosis was based on histopathological findings (8 core biopsies, 2 liver resections). The detection of one or more IPTLs on imaging was accidental in 8 cases; in two cases, imaging was performed for patient symptoms (1 palpable abdominal mass, 1 fever and upper abdominal pain): 5 patients had associated conditions and the other 5 (50%) were free of any disease at the time of IPTL detection. In 3 patients (30%), hepatic steatosis was detected. Two out of 10 patients had IGg4-positive plasma cell infiltrate at pathology (males, with a solitary nodule); in these, also IgG4 serum levels were increased (> 135 mg/dL). A total of 16 IPTLs were detected: single in 8 cases and multiple in 2 cases (2 and 6 nodules). The mean maximum diameter of the IPTLs was 40 mm (size range 15–200 mm). Epidemiological and clinical features and literature comparisons are summarized in Table 2.

### Lesions’ location and their relationship with bile and blood vessels

In literature the segment site is known for 34 IPTLs for a total of 42 occupied segments, as reported (Table 3). The lobe site is known for 113 IPTLs, comprehensive of the latter 34 IPTLs with known segment site. Then, 46 (40.7%) and 67 (59.3%) IPTLs were found in the left and right lobe, respectively. Regarding possible relation with the bile ducts, we have found some information in 9 out of the selected 38 papers [7, 8, 10, 16, 21, 27, 32, 36, 41]. The IPTL determines prevalently biliary ectasia, but in two papers, a small ducts disruption adjacent to the mass [27] or lithiasis with signs of cholangitis is documented [36]. About potential damage of blood vessels determined by IPTL, data are really few, only 4 papers out of 38. The most relevant series is regarding “blood vessel penetration” of 14 out of 31 (45.2%) IPTLs [7]. Moreover, a displacement of inferior vena cava without invasion [24], a thrombosis of the right hepatic vein [29], and an occlusion of afferent portal branch [44] are reported. In our series, 16 IPTLs were detected, with a total of 22 occupied segments; 8 (38.1%) in the left and 14 (61.9%) in the right lobe (Table 3). Regarding the relationship with bile ducts and blood vessels, most of the IPTLs of our series do not cause alterations. A “blood vessel and biliary duct penetration”- a behavior we can better define as “insinuative” (both biliary and blood vessels) was detected in 2 of our cases.

**Table 3:** Localization of IPTLs in literature and our experience

	S1 *n* (%)	S2 *n* (%)	S3 *n* (%)	S4 *n* (%)	S5 *n* (%)	S6 *n* (%)	S7 *n* (%)	S8 *n* (%)
Literature								
34 IPTLs occupying 42 segments	2 (4.8%)	3 (7.1%)	3 (7.1%)	11 (26.2%)	5 (11.9%)	8 (19.0%)	3 (7.1%)	7 (16.8%)
Our experience								
16 IPTLs occupying 22 segments	0 (0%)	3 (13.6%)	4 (18.2%)	1 (4.5%)	0 (0%)	2 (9.1%)	6 (27.3%)	6 (27.3%)

Legend: *n* (%): number and percentage of occupied hepatic segments (S)

If the localization of IPTL extends to more than one segment, all the segments mentioned in the text were considered (see also the text). So, the segments occupied result more than the number of IPTLs

### Patterns of lesions SI

All possible imaging patterns found in the literature regarding T1/T2W images, CE-T1W images, HBP, and DWI (data available for 68, 113, 27 and 40 IPTLs, respectively), with the relative percentages of presentation, are summarized in Table 4 (bolded fonts). Great variability in T1/T2 SI on a per-lesion basis was observed, although multiple IPTLs in the same liver showed similar imaging features. Most of the lesions presented homogeneous T1 hypointensity (88%, 60/68 of IPTLs); homogeneous/inhomogeneous T2 hyperintensity (60%, 40/68) or T2 two-layered concentric “targetoid” appearance with hyperintense central core (31%, 21/68); a two- or three-layered concentric targetoid enhancement pattern visible at least in one phase of the dynamic study (51%, 57/113), mainly with a hypointense central core (42%, 47/113); and a two-layered targetoid pattern with a hyperintense peripheral rim on HBP (63%, 17/27) and homogeneous hyperintensity on high *b*-values (> 750 mm^2^/s) on DWI (82%, 33/40). A more detailed description is reported in Supplemental 2.

**Table 4:** Pattern of Magnetic Resonance features

	Definitely hypointense (%)	Definitely hyperintense (%)	Isointense (%)	Inhomogeneous at unenhanced images or mosaic enhancement (%)	Targetoid with hypointense core (%)	Targetoid with hyperintense core (%)
T1	**88** *25*	**6** *0*	–	–	**4.5** *63*	**1,5** *12*
T2	**8** *0*	**35** *44*	–	**25** *12*	–	**31** *44*
DYN-CE T1	**8** *0*	Progressive enhancement **13** *6.5*	–	**27** *12.5*	**42** *81*	**9** *0*
HBP	**33** *100*	–	–	–	**63** *0*	**4** *0*
DWI	–	**82** *0*	–	–	**18** *100*	–

Bolded fonts: literature. Italics fonts: our experience. Progressive enhancement: slow enhancement from arterial to equilibrium phase

*DYN* dynamic, *CE* contrast enhanced, *HBP* hepatobiliary phase, *DWI* diffusion-weighted imaging

**Fig. 3:**
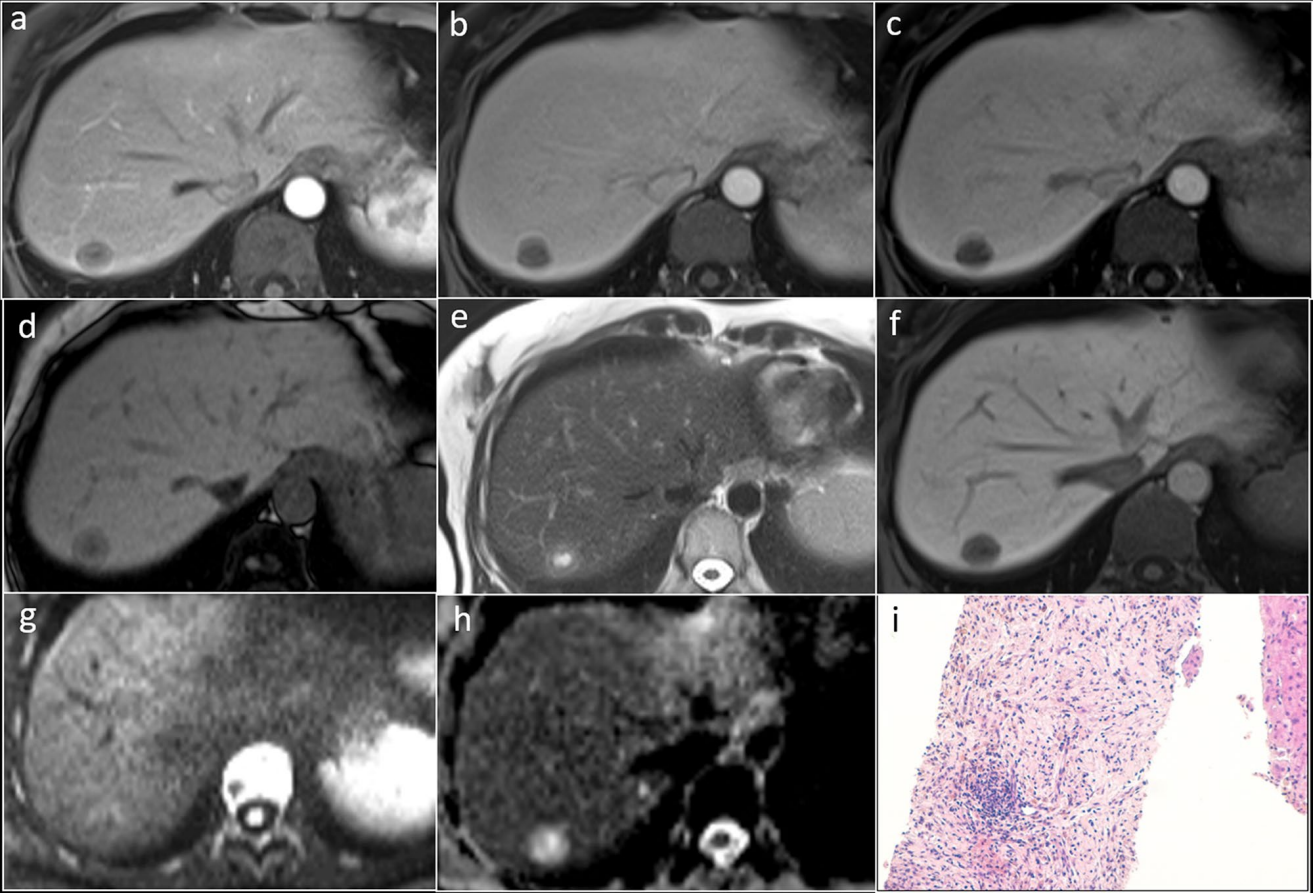
Inflammatory pseudotumor of the liver of the hepatic segment 7 in patient with history of breast cancer, 9 years ago. On MR, two-layered concentric “targetoid” appearance with the hypointense central core is seen on arterial phase (**a**) and gradient echo T1W out of phase (**d**). Target appearance is maintained on T2W images (**e**), with hyperintense central core, high *b*-value (750 mm^2^/s) DWI (**g**), and ADC map (**h**) with low and high central core signal intensity, respectively. On portal (**b**), equilibrium (**c**), and hepatobiliary phase (**f**), the lesion shows signal hypointensity. A transcutaneous biopsy was performed. Histological examination (**i**, hematoxylin–eosin, original magnification × 100) shows a mixture of spindle-shaped cells (myofibroblasts and fibroblasts) and inflammatory cells (predominantly plasma cells and lymphocytes with scattered neutrophils and eosinophils). The findings are indicative of inflammatory pseudotumor

All imaging patterns found in our experience and relative percentages of presentation are shown (Table 4—italics fonts). Even in our experience, great variability in T1/T2 SI on a per-lesion basis was observed, although multiple IPTLs in the same liver showed similar imaging features. Most of the lesions presented a two- or three-layered concentric “targetoid” appearance on T1W images (63%, 10/16 of IPTLs, with the hypointense central core); homogeneous/inhomogeneous T2 signal hyperintensity (56%, 9/16) or targetoid T2 appearance with a hyperintense central core (44%, 7/16); a two- or three-layered concentric target enhancement pattern at least in one phase of the dynamic study (81%, 13/16; this latter pattern associated in half of the cases with a T2 targetoid appearance); homogeneous signal hypointensity on HBP, and a two-layered targetoid appearance on DWI high *b*-values (> 750 mm^2^/s) with a core of low SI and a peripheral hyperintense halo in all cases (100%, 16/16) (Fig. 3). Targetoid appearance with peripheral hypointense halos and hyperintense cores was observed in 5 IPTLs on ADC maps; the other 11 IPTLs had ADC values always equivalent to or higher than those of the surrounding parenchyma. A more detailed description is reported in Supplemental 3.

### Lesion SI follow-up

In the literature, we found documentation of at least one follow-up (maximum of 12 months) with MRI in 24 IPTLs, disappearing in 7 cases (29%, after steroid therapy, with ursodeoxycholic acid or unspecified) and persistent in the remaining 17 (71%). Persistent IPTLs tend to maintain the T1 SI of the first MR examination. Only three T1W homogenous hypointense IPTLs evolved in a T1W two-layered concentric targetoid appearance with a hypointense central core. On T2W images, there is a clear tendency to evolve in the homogeneous hypointense pattern, regardless of the starting hypo or hyper SI. On CE images, a double tendency is observed: either an evolution toward a hypovascular (homogenous hypointense) pattern or maintenance of the starting pattern. At the DWI follow-up (very few cases and always without ADC data), there was a tendency toward the loss of cellular crowding over time: two IPTLs evolved into homogeneous hypointense lesions and one evolved into a two-layered targetoid pattern with a hyperintense rim and hypointense core.

**Fig. 4:**
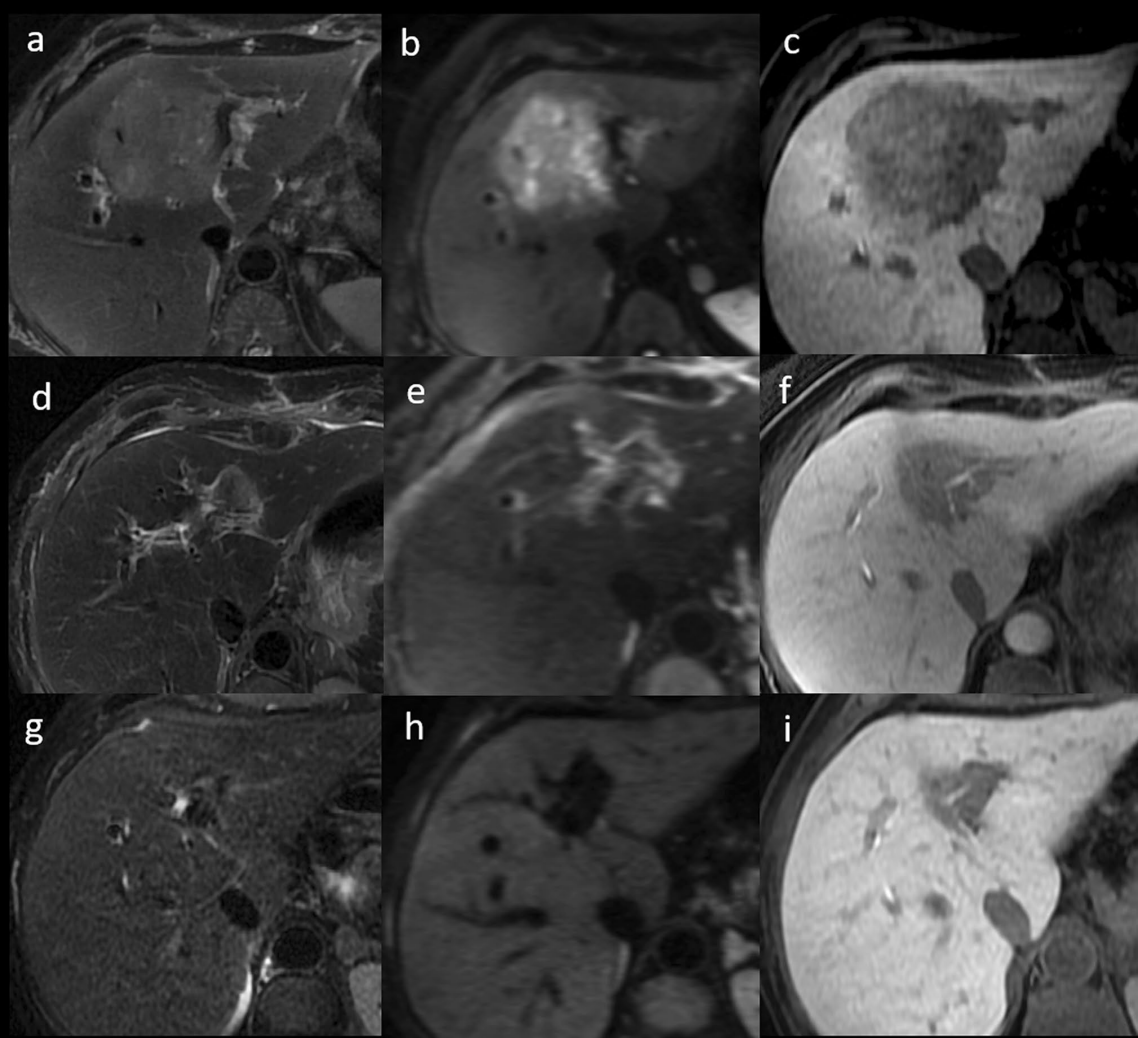
Rapid regression of incidental inflammatory pseudotumor of the liver (IPTL) in a 67-year-old Caucasian woman. MR was performed after US examination for fever and abdominal pain in March 2012, detecting a large hypoechoic lesion of the liver (not shown). MR confirmed the lesion with inhomogeneous signal hyperintensity on fat sat T2W (**a**) and high *b*-value DW (**b**) images, hypointensity on hepatobiliary phase (**c**). With the histological diagnosis of IPTL (mixed inflammatory infiltrate and spindle cells on needle biopsy whip), corticosteroid therapy was undertaken at the beginning of April 2012. This allowed a subtotal regression of the lesion as early as May 2012, as shown on T2W (**d**), DW (**e**), and hepatobiliary phase (**f**). On the same sequences (**g**–**i**), a complete regression is seen on MR follow-up in September 2013

**Fig. 5:**
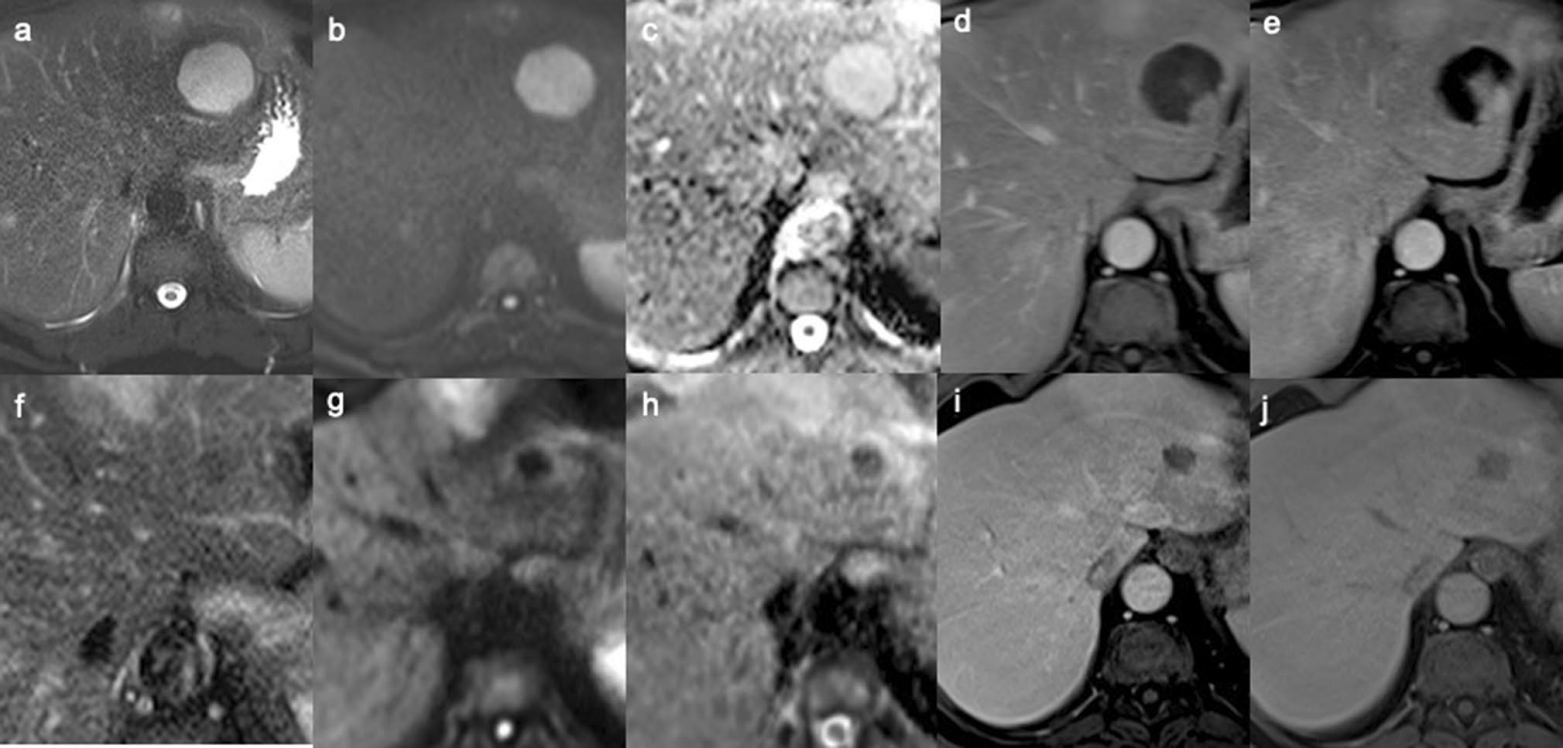
Histologically proven inflammatory pseudotumor of the liver (IPTL) in the left hepatic lobe during MR follow-up in patient with focal nodular hyperplasia (partially visible in front of IPTL). In 2017, at the onset, IPTL shows high hydration in T2W (**a**), high *b*-value DW (**b**) images, and in the ADC map (**c**). Progressive inhomogeneous enhancement at peripheral starting is shown in the portal (**d**) and equilibrium (**e**) phases. After 24 months, size reduction of the lesion, T2 (**f**) signal hypointensity, spin mobile lessening on DW images (**g**) and ADC map (**h**), and hypovascularization in the portal (**i**) and equilibrium (**j**) phases are observed. On histological examination the main cell population is macrophages, intermixed with other chronic inflammatory cells, like plasma cells and lymphocytes. Some scattered hepatocytes are sequestered in the inflammatory infiltrate. S100 and CD1a stains: negative. Endothelial markers: negative. The findings indicate as the more likely the diagnosis of inflammatory pseudotumor

In our experience, 8 patients out of 10 with 8 IPTLs underwent at least 1 follow-up with MRI (between 3 and 12 months after baseline). Lesions were no longer detectable at the first follow-up MRI in 4 cases (each with a single nodule) or at the second follow-up MRI in 2 cases (each with a single nodule; between 6 and 36 months from the previous control), always after steroid therapy (Fig. 4). In the remaining 2 patients (8 lesions total; 6 lesions in one patient, two in the other), the persistence of lesions was documented in the two subsequent MRI follow-ups. On T1W images, persistent IPTLs maintained a “targetoid appearance” with a central hypointense core; only 1 lesion progressed from a “targetoid” with a hyper to hypointense core. On T2W images, persistent IPTLs always evolved into homogeneously hypointense lesions. In CE dynamic phases, persistent IPTLs evolved from “targetoid” enhancement into homogeneously hypointense and hypovascular lesions. On DWI, persistent IPTLs evolved into homogeneously hypointense lesions in all our cases, with a homogeneous low SI on the ADC map (Fig. 5).

## Discussion

Both the literature analysis and the series we propose *confirm the polymorphism of IPTL*. US, CT, and MR imaging aspects of this disease cause IPTL to be frequently misdiagnosed with a primary or secondary liver malignancy or abscess [46]. Contrast-enhanced US assessment of IPTL also exhibits great variability: some studies defined rapid arterial phase hyper-enhancement and wash-out in the portal phase as the main contrast-enhanced US feature of IPTL [47], which would make differential diagnosis (DD) impossible with malignancies, while others highlighted hypo-enhancement in the arterial/portal phase and iso-enhancement in the delayed phase [48], which conversely would allow easier distinction from a malignancy. Still, others showed the absence of enhancement in part or all of the IPTLs, probably due to coagulative necrosis [49]. Fluorodeoxyglucose-positron emission tomography can also induce the diagnosis of malignancy due to the often high glucose uptake of IPTL [12]. When more nodules are present, it is possible to find a different glucose uptake by each nodule due to the different temporal stages of each nodule [50].

**Fig. 6:**
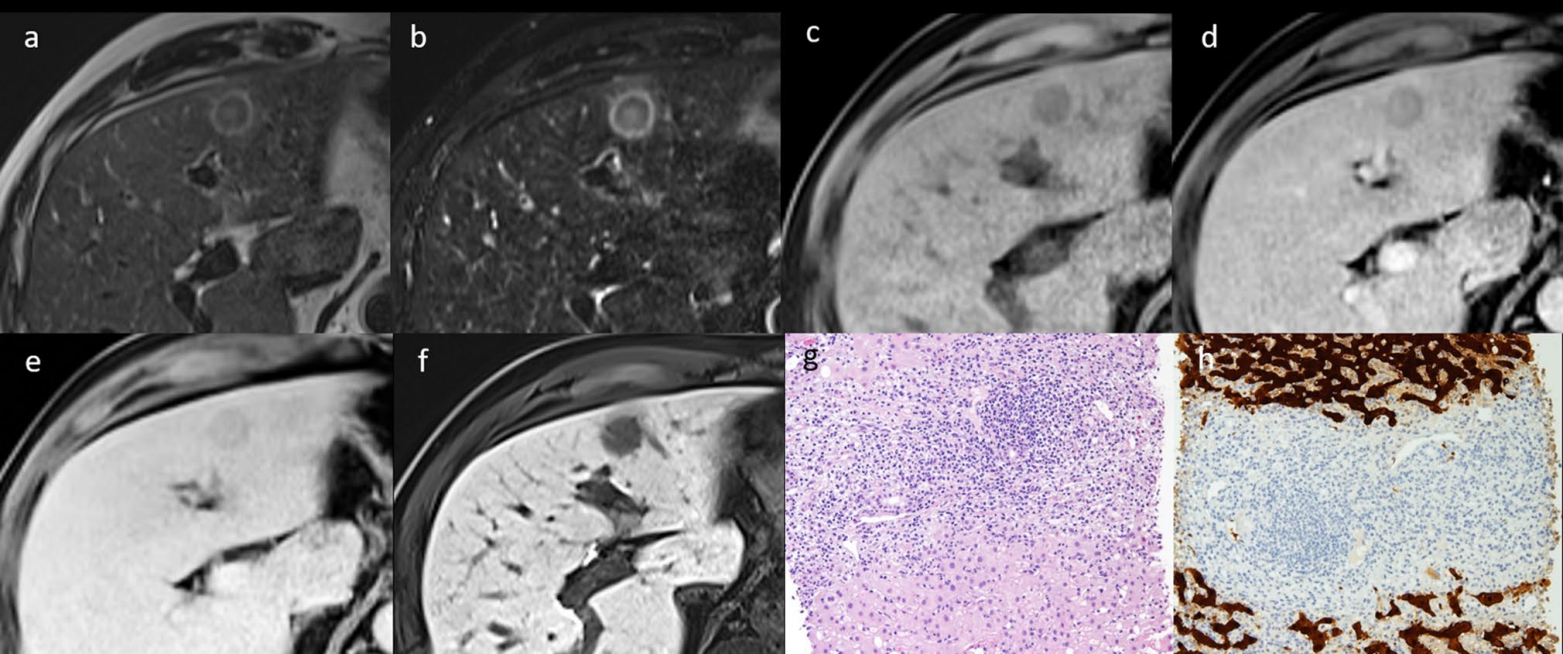
IgG4-related inflammatory pseudotumor of the liver of a 58-year-old Caucasian male patient. MR T2W (**a**) and T2 SPAIR (**b**) images show a three-layered concentric targetoid aspect lesion with hyperintense core. CA administration confirms the targetoid aspect of the lesion on arterial (**d**) and portal (**e**) phase. On unenhanced T1W (**c**) and hepatobiliary phase (**f**), the lesion appears hypointense. Pathologic analysis of needle biopsy whip shows inflammatory infiltrates with polyclonal cells, myofibroblastic-fused cells, eosinophilic granulocytes, and band of fibrosclerotic tissue (**g**, hematoxylin–eosin stain, original magnification × 100). On IgG4 immunostaining (not shown) IgG4-positive plasma cells > 20/HPF. On Arginasi 1 coloration (**h**, × 40) infiltrates inflammatory cells (median area) with residual biliary duct, surrounded by normal hepatic parenchyma (upper and lower areas, brown colored) are shown

*There are no specific epidemiological or clinical features of IPTL*. It more commonly affects young adults, with a higher incidence in Asian countries, but can occur at any age [3]. We did not find the most frequent associated diseases (sclerosing cholangitis, retroperitoneal fibrosis, and autoimmune diseases) [3] but other non-specific conditions, such as chronic/fatty liver disease and history of neoplasia (rectal cancer or lymphoma). According to previous works, clinical symptoms (upper abdominal pain, fever, jaundice, asthenia, palpable abdominal mass) would also be helpful but non-specific for the diagnosis of IPTL [2, 3]. Overall, in the 51/124 patients for whom the data were available, the presence of IgG4-positive plasma cells in the inflammatory infiltrate of IPTL was detected in 21 patients (41%). According to our analysis, the most common presentation is a solitary nodule (Fig. 6) [51]. A typical histological pattern of IgG4-related IPTL shows abundant IgG4-positive plasma cell infiltration (> 10 cells/HPF in biopsy and > 50 cells in surgical materials), fibrosis, and obliterative phlebitis [52]. In the literature, high-serum IgG4 levels (> 135 mg/dL) are reported in more than 85% of cases of IgG4-related IPTL [51]; similarly, in two of the cases by us, IgG4 related. On this basis, the search for positivity for IgG4 in case of a radiological suspicion of IPTL appears useful to direct the patient’s appropriate treatment and possibly avoid biopsy. It is not possible to exclude the “old” hypothesis according to which, with the temporal evolution of IPTL, from the plasma cell granuloma to the xanthogranuloma and sclerosing type, the IgG4 lymphocyte response could fail and therefore the IgG4 serum levels and infiltrate are lost [53].

**Fig. 7:**
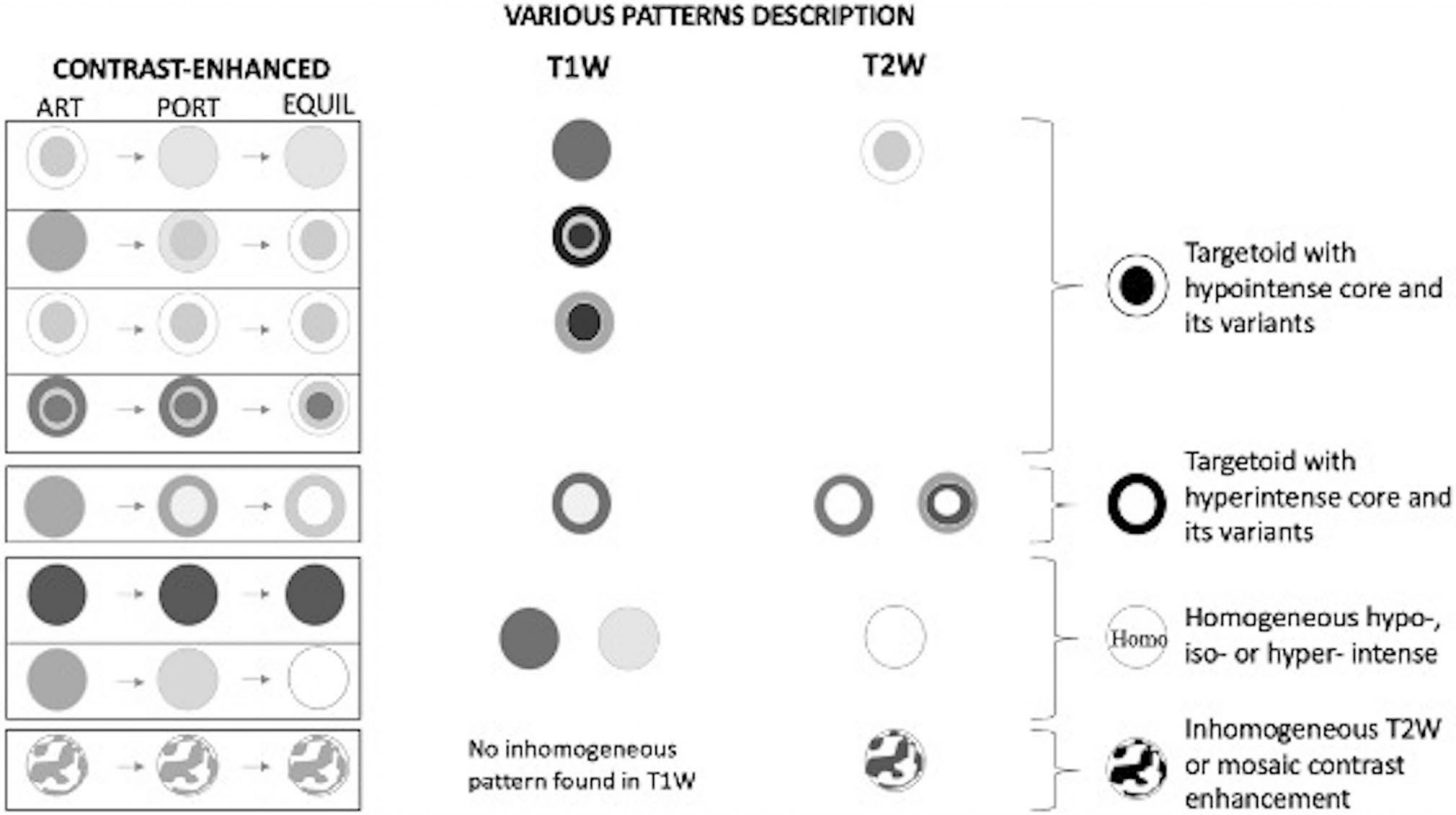
MR patterns of inflammatory pseudotumors of the liver found both in literature and our experience

Although there are differences between the literature and our series, *some reproducible and rather typical imaging findings for IPTL are confirmed*. Considering the MR SI pattern (Table 4), both in the literature and in our series, on T2W images, the dominant patterns are homo/inhomogeneous signal hyperintensity (60% vs. 56%, respectively) and targetoid appearance with a hyperintense core (31% vs. 44%). Differences between the literature results and our series are more evident on T1W images, where the lesions mostly appear homogenously hypointense in the literature review (88% of the lesions) and targetoid with a hypointense core in our group of patients (63% of the lesions). However, these differences became less evident after CA administration, where the IPTLs most frequently showed a three- or two-layered concentric targetoid aspect with cores of varying SI (51% vs. 81% for literature vs. our series, respectively), mosaic enhancement (27% vs. 12.5%), and homogeneous progressive enhancement (13% vs. 6.5%). In the HBP, either in our experience (16 IPTLs) or in the literature (27 IPTLs), the only pattern is the hypointense one, confirming the absence of functioning hepatocytes; in the literature, a hyperintense peripheral rim is often described, which we believe may be attributable to compressed liver parenchyma or interstitial CA permeation phenomena. All the possible MRI patterns found in the literature and our cases on T1/T2W and dynamic CE images are summarized in Fig. 7. On DWI, targetoid appearance at high b-values is reported in 18% of IPTLs in the literature data (7/40 lesions) vs. 100% of our series (16/16 lesions). Literature data about the ADC map are not available, while in our cases, the map values ​​were always equal to or higher than those of the surrounding parenchyma. The target aspect of IPTL, in summary, is very frequent in our experience (75% on T1W, 44% on T2W, 81% on CE-T1W (at least one phase), 100% on DW images) but is also recurrent in the literature (6% on T1W, 31% on T2W, 51% on CE-T1W (at least one phase), 18% on DW images, and 67% on HBP).

As regards the location of IPTLs in the liver, both for literature and our series is about 40 and 60% for left and right lobe, respectively. Thus, bearing in mind the volumetric differences of the two lobes, we can believe that there is no preferential localization of IPTLs in the liver parenchyma. When very close to biliary vessels, IPTL can rarely determine biliary ectasia, but in two papers, a small ducts disruption [27] or lithiasis and cholangitis are documented [36]. About potential abutment, encasement, and occlusion of blood vessels determined by IPTL, the most interesting data are regarding “blood vessel penetration” of 14 out of 31 (45.2%) IPTLs [7]. This behavior, better defined as “insinuative” (both biliary and blood vessels), was detected also in 2 of our cases (see Fig. 4). Interestingly, this insinuative pattern has been reported also in some primitive hepatic lymphomas [54] and so, this similarity could help us to explain why sometimes IPTLs were defined “pseudo-lymphomas.”

*With regards to the DDs*, not due in a systematic review, but in our opinion useful in this context, focal nodular hyperplasia with a great central scar can create some diagnostic problems against a targetoid-shaped IPTL with a hypointense core. However, after liver-specific CA administration, the diagnosis is easy either for strong, homogeneous, arterial phase hyper-enhancement or for features of the HBP of FNH [2, 55, 56]. A pyogenic abscess usually presents as a multiseptate mass with unenhancing contents, sometimes with an enhancement ring due to compressed liver parenchyma [57]. Fungal collections are probably the more difficult DD. Untreated, subacute post-treatment, and chronic healed fungal hepatic abscesses usually appear as multiple, small lesions; the targetoid aspect could be maintained on unenhanced imaging, but fungal balls show only a slight and inconstant CE. However, a history of myelosuppressive therapy and the multiplicity of the lesions are very important for a correct diagnosis [57]. Sometimes hepatic peliosis may show a targetoid aspect. However, the DD can be performed on T2W images, where peliosis is commonly seen as a very high SI lesion (similar to hemangioma). After CA administration, peliosis can show centripetal or centrifugal enhancement with a completely different pattern with respect to IPTL [58]. Considering malignant focal liver lesions, hepatic epithelioid hemangioendothelioma frequently shows a target appearance on T1/T2/DW images, a ring or target enhancement pattern on the dynamic study, and low enhancement at the HBP, similar to IPTL. However, hepatic epithelioid hemangioendothelioma more often shows a peripheral distribution of nodules (often multiple), with coalescence and capsular retraction [59]. Some atypical IPTLs may present with arterial phase hyper-enhancement and are difficult to differentiate from hepatocellular carcinomas. Peripheral cholangiocarcinoma might have the imaging features of a heterogeneous mass with delayed enhancement; peripheral biliary duct dilatation, thickening of the bile ducts, and the presence of lymphadenopathy are typical signs [60]. Last, despite recent progress in the diagnostic ability of imaging, it is difficult to differentiate IPTL from metastases. These can show the “doughnut sign” due to a necrotic center and surrounding viable tumor with central T1/T2 hypo/hyperintensity and peripheral T1/T2 hyper/hypointensity [57]. Moreover, all metastatic lesions, whether hypovascular or hypervascular, demonstrate peripheral arterial ring enhancement because of peritumoral desmoplastic reaction [57].

*Close follow-up is frequently essential for the diagnosis* *of IPTL*, because it tends to fade out over time. In our analysis, IPTL disappeared in 7/24 of the literature’s follow-ups and 6/8 of our case’s follow-ups. The higher number of patients showing persistence of the nodules in the literature analysis may be related to the shorter duration of the follow-up (maximum of 12 months vs. 36 months). Persistent IPTLs evolved, as a whole, into lesions with progressive lower SI on T2W images, up to homogeneous T2 hypointensity, regardless of the starting pattern, with hypo- or hyper-T2 SI. Therefore, imaging is indicative, at the regression of injury, of a decrease in the state of hydration, similar to the coagulative necrosis pattern. DWI confirms this evaluation: low SI on high b-value DW images and ADC maps were observed in all persistent IPTLs of our population, outlining the so-called “spin mobile lessening” and so a very low water content (Fig. 5) [61]. This result seems to validate the orientation of those pathologists who retain the scleroyalin form, with lower watery content and the evolution of the other plasma cell and xanthogranuloma types [53]. Finally, in our opinion, this tendency to evolve (and then to be involved) over time strengthens the polymorphism of IPTL.

*This work has some limitations *both for literature data and our series. Regarding the literature, it must be emphasized that we followed the applicable PRISMA items and proposed a systematic review, but not a meta-analysis [62]. The data found in literature were very fragmented as most of the papers were Case Reports. For this reason, the final analysis involves large discrepancies in the features presented; moreover, the few and short follow-ups of the lesions allow only a partial evaluation of the IPTLs found in literature over time. The few series available [7, 8, 31, 45] provided more information but only one [19] fully followed Munn’s criteria. However, considering the low disease prevalence, based on this study, can it be hypothesized that the prevalence is underestimated due to the tendency of IPTL to fade out over time? It would be complex to plan a prospective study for which, where possible, our results can represent the starting point. Another limitation could be seen in the difference between the races of the literature cases, 91% Asiatic, and that of our cases, all Caucasian. Finally, although it might be asserted “no novelty from this work,” it is the first attempt to organize the fragmented literature knowledge in one paper, with the integration of 10 MR case series, which represents one of the major MR series in the literature, with 10/10 case complete of histological findings and 7/10 cases followed over time for over 3 years.

*In conclusion*, our work confirms the IPTL polymorphism, but it also demonstrates that when focal liver lesions present with a “targetoid-like” aspect on T1–T2W images, with hypointensity on the HBP and ADC values higher than that of the surrounding parenchyma, the diagnosis of IPTL must be considered, particularly if IgG4-positive plasma is detected. This pattern is not pathognomonic, so it should drive (with or without core biopsy, as appropriate on the background of the clinical data of the patient) to steroid therapy and follow-up, which can demonstrate lesion disappearance or involution in a dehydrated pattern resembling coagulative necrosis.

## Supplementary Information

Below is the link to the electronic supplementary material.

Supplementary file1 (DOCX 24 kb)

## Abbreviations

IPTL: Inflammatory pseudotumor of the liver
MRI: Magnetic resonance imaging
US: Ultrasound
CT: Computed tomography
DWI: Diffusion-weighted imaging
T1/T1W: T1/T2 weighted
CE: Contrast enhanced
ADC: Apparent diffusion coefficient
CA: Contrast agent
HBP: Hepatobiliary phase
SI: Signal intensity
