# Supplementary material for: Magnetic resonance imaging of inflammatory pseudotumor of the liver: a 2021 systematic literature update and series presentation
Source: Abdom Radiol (NY). 2022 Jun 1;47(8):2795–810. doi: 10.1007/s00261-022-03555-9 (PMC9300573; doi:10.1007/s00261-022-03555-9)
Supplement: Supplementary file 1 — Supplementary file1 (DOCX 24 kb) [file 261_2022_3555_MOESM1_ESM.docx]

**Supplementary materials**

**Supplementary 1**

**Quality Assessment tables**

**Quality assessment – Case reports**

How to cite: Moola S, Munn Z, Tufanaru C, Aromataris E, Sears K, Sfetcu R, Currie M, Lisy K, Qureshi R, Mattis P, Mu P. Chapter 7: Systematic reviews of etiology and risk. In: Aromataris E, Munn Z (Editors). JBI Manual for Evidence Synthesis. JBI, 2020. Available from https://synthesismanual.jbi.global. https://doi.org/10.46658/JBIMES-20-08

|  | Were patient’s demographic characteristics clearly described? | Was the patient’s history clearly described and presented as a timeline? | Was the current clinical condition of the patient on presentation clearly described? | Were diagnostic tests or assessment methods and the results clearly described | Was the intervention(s) or treatment procedure(s) clearly described? | Was the post-intervention clinical condition clearly described? | Were adverse events (harms) or unanticipated events identified and described? | Does the case report provide takeaway lessons? |
| --- | --- | --- | --- | --- | --- | --- | --- | --- |
| *Hayashi, 2021* | YES | UNCLEAR | YES | YES | YES | YES | NA | YES |
| *Wang, 2020* | YES | NO | UNCLEAR | YES | YES | NO | NA | YES |
| *Hamano, 2020* | YES | YES | YES | YES | YES | YES | NA | YES |
| *Kim, 2020* | YES | YES | YES | YES | YES | YES | NA | YES |
| *Lameirão G, 2018* | YES | YES | YES | YES | YES | YES | NA | YES |
| *Pantiora, 2018* | YES | YES | YES | YES | NO | NO | NA | YES |
| *Miyajima, 2018* | YES | YES | YES | YES | YES | YES | NA | YES |
| *Fuchino, 2017* | UNCLEAR | YES | YES | YES | YES | YES | NA | YES |
| *De Mel, 2016* | YES | YES | YES | YES | YES | NO | NA | YES |
| *Bae, 2015* | UNCLEAR | NO | YES | YES | YES | NO | NA | YES |
| *Obana, 2015* | UNCLEAR | UNCLEAR | YES | YES | YES | YES | NA | YES |
| *Al-Hussaini, 2015* | YES | YES | YES | YES | YES | YES | NA | YES |
| *Chang, 2014* | YES | YES | YES | YES | YES | YES | NA | YES |
| *Matsuo, 2014* | YES | YES | YES | YES | YES | YES | NA | YES |
| *Durmus, 2014* | UNCLEAR | YES | YES | YES | YES | NO | NA | YES |
| *Iguchi, 2013* | YES | YES | YES | YES | YES | YES | NA | YES |
| *Ke, 2013* | UNCLEAR | UNCLEAR | YES | YES | YES | NO | NA | YES |
| *Shirai, 2013* | UNCLEAR | YES | YES | YES | YES | YES | NA | YES |
| *Rosa, 2012* | YES | UNCLEAR | YES | YES | YES | YES | NA | YES |
| *Jeong, 2012* | YES | YES | YES | YES | NO | YES | NA | YES |
| *Saito, 2012* | YES | YES | YES | YES | YES | YES | NA | YES |
| *Herek, 2011* | UNCLEAR | NO | YES | YES | UNCLEAR | YES | NA | NO |
| *Ueda, 2009* | UNCLEAR | UNCLEAR | YES | YES | YES | YES | NA | NO |
| *Ganesan, 2009* | UNCLEAR | NO | YES | YES | YES | YES | NA | YES |
| *Motojuku, 2008* | UNCLEAR | UNCLEAR | YES | YES | YES | NO | NA | YES |
| *Vassiliadis, 2007* | UNCLEAR | YES | YES | YES | YES | YES | NA | YES |
| *Teranishi, 2005* | UNCLEAR | NO | YES | YES | YES | YES | NA | YES |
| *Alimoglu, 2005* | UNCLEAR | NO | YES | YES | YES | YES | NA | YES |
| *Kato, 2004* | UNCLEAR | NO | YES | YES | YES | NO | NA | YES |
| *Schneider, 2003* | UNCLEAR | NO | YES | YES | UNCLEAR | YES | NA | YES |
| *Saito, 2002* | UNCLEAR | NO | YES | YES | YES | NO | NA | YES |
| *Mortelé, 2001* | UNCLEAR | UNCLEAR | YES | YES | YES | YES | NA | YES |
| *Sakai, 2001* | UNCLEAR | UNCLEAR | YES | YES | YES | YES | NA | YES |
| *Toda, 2000* | UNCLEAR | NO | YES | YES | YES | YES | NA | YES |
| Answers: Yes, No, Unclear or Not/Applicable | | | | | | | | |

**Quality assessment - Cross sectional studies**

How to cite: Munn Z, Moola S, Lisy K, Riitano D, Tufanaru C. Methodological guidance for systematic reviews of observational epidemiological studies reporting prevalence and incidence data. Int J Evid Based Healthc. 2015;13(3):147–153.

|  | Was the sample frame appropriate to address the target population? | Were study participants sampled in an appropriate way? | Was the sample size adequate? | Were the study subjects and the setting described in detail? | Was the data analysis conducted with sufficient coverage of the identified sample? | Were valid methods used for the identification of the condition? | Was the condition measured in a standard, reliable way for all participants? | Was there appropriate statistical analysis? | Was the response rate adequate, and if not, was the low response rate managed appropriately? |
| --- | --- | --- | --- | --- | --- | --- | --- | --- | --- |
| *Ichikawa, 2020* | UNCLEAR | YES | YES | YES | YES | YES | YES | YES | YES |
| *Sheng, 2017* | UNCLEAR | YES | YES | YES | YES | YES | YES | YES | YES |
| *Park, 2014* | YES | YES | YES | YES | YES | YES | YES | YES | YES |
| *Yan, 2001* | UNCLEAR | YES | YES | YES | YES | YES | YES | YES | YES |

**Supplementary 2**

**Literature Data - Patterns of lesions** **SI**

T1 and T2 SI. A great variability in T1/T2 SI on a per-lesion basis was observed, although multiple IPTLs in the same liver showed similar imaging features. In T1W images the great majority of lesions (88%, 60/68 of IPTLs) showed a homogenous hypointense appearance. Other T1W patterns (homogenous hyperintense 6%, three- and two-layered concentric “targetoid appearance” [with hypointense or hyperintense core] 6%) are a minority. In T2W images, the most frequent pattern was homogenous hyperintense (35%, 24/68 of IPTLs), followed by "targetoid" appearance with hyperintense central core (31%, 21/68) and "inhomogeneous" pattern with hyperintense alternating with hypointense areas without a defined distribution (25%, 17/68). The homogeneously hypointense pattern in T2W at diagnosis was the least frequent, found in 8% of cases (6/68).

Contrast-Enhanced Studies. Considering the contrast-enhanced MRI, conventional Gd-chelates were used in eighty-six patients while, in twenty-seven patients a liver-speciﬁc Gd-chelates contrast agent was used. Multiple IPTLs in the same liver showed a similar pattern. Considering the overall enhancement pattern, 51% (57/113) of patients showed a “targetoid” enhancement pattern visible at least in one phase of the dynamic study. Of this "targetoid" pattern, the vast majority, forty-seven (42%), show a hypointense central core, only 10 (9%) a central core enhancement. Fifteen patients (13%) showed a homogeneous and progressive enhancement pattern, thirty-two (27%) patients showed a heterogeneous enhancement pattern. Nine patients (8%) showed at diagnosis a homogenous hypointense (hypovascular) pattern.

Hepatobiliary Phase. At hepatobiliary phase, that was available in twenty-seven patients, the majority of IPTLs (63%, 17/27) showed a two-layered targetoid pattern with hyperintense peripheral rim; nine IPTLs showed a homogeneous low SI with respect to the surrounding healthy (33%). Only one case (4%) showed a two-layered targetoid pattern with hyperintense central core.

Diffusion-Weighted imaging. DWI was available in forty IPTLs. Data on b-values ​​and other diffusion parameters were not available in all cases. However, only DW images on high b-values (>750 mm2/s) were considered to derive the DWI pattern. ADC maps have never been reported in the literature for IPTL. The most frequent DWI pattern is represented by the homogeneous hyperintense (82%; 33/40); seven IPTLs (18%) showed a two-layered targetoid pattern with hyperintense peripheral rim.

**Supplementary 3**

**Our experience - Patterns of lesions** **SI**

T1 and T2 Signal. A great variability in T1/T2 SI on a per-lesion basis was observed, although multiple IPTLs in the same liver showed similar imaging features. The majority of lesions (63% of IPTLs, 10/16, with 6 multiple identical nodules in the same patient) showed a “targetoid appearance” on T1W images (a three- and two-layered concentric pattern always with the hypointense central core). Four cases (25%) had a hypointense homogeneous appearance on T1W images and two cases (12,5%) a "targetoid appearance" but with a hyperintense central core. On T2W images, seven cases (44%) were homogeneously hyperintense, seven (44%) with "targetoid" appearance. The targetoid appearance in T2W were supported by a hyperintense or iso/slightly hyperintense central core. Two cases (12%) showed a T2 "heterogeneous" pattern with hyperintense areas alternating with hypointense areas without a defined distribution.

Contrast-Enhanced Studies. In all patients a liver-speciﬁc Gd-chelates contrast agent was used (Gd-EOB-DTPA in all cases). Different patterns of enhancement were observed among IPTLs of our series, but IPTLs in the same liver showed a similar pattern. Considering the overall enhancement pattern, 81% (13) of our lesions showed a targetoid enhancement pattern (either ring-enhancement or target-enhancement pattern) visible at least in one phase of the dynamic study. One lesion (6,5%) showed a homogeneous and progressive enhancement pattern, two (12,5%) lesions showed a heterogeneous enhancement pattern. In our series 6/13 IPTLs having a targetoid enhancement pattern showed a T2 targetoid appearance. On the other hand, only one pseudotumor showed a targetoid appearance on T2W images (given by a large central colliquative area) without the targetoid dynamic enhancement pattern.

Hepatobiliary Phase. Hepatobiliary phase was available in all patients (ten IPTLs) of our series. At hepatobiliary phase IPTLS of our series showed a homogeneous low SI with respect to the surrounding healthy liver in all patients (100%).

Diffusion-Weighted imaging. DWI was available in all patients (seven IPTLs). In this series b values of 0 and 750 mm^2^/sec and ADC maps were available in all patients. At b 750 mm^2^/sec IPTLs of our series showed a two-layered targetoid appearance with a core of low SI and a peripheral hyperintense halo in all cases (100%). The targetoid appearance of IPTL at b 750 mm^2^/sec corresponded to a ring appearance on the ADC map (peripheral hypointense halo, and high SI of the core lesions) in 5 IPTLs.
